# Effectiveness of α-, γ- and δ-Tocopherol in a CLA-Rich Oil

**DOI:** 10.3390/antiox3010176

**Published:** 2014-03-17

**Authors:** Gloria Márquez-Ruiz, María del Carmen García-Martínez, Francisca Holgado, Joaquín Velasco

**Affiliations:** 1 Instituto de Ciencia y Tecnología de Alimentos y Nutrición (ICTAN-CSIC), José Antonio Novais 10, Madrid 28040, Spain; E-Mails: bmc_mary@yahoo.es (M.C.G.-M.); pha@if.csic.es (F.H.); 2 Instituto de la Grasa (IG-CSIC), Padre García Tejero 4, Sevilla 41012, Spain; E-Mail: jvelasco@cica.es

**Keywords:** antioxidants, CLA, conjugated linoleic acid, functional lipids, lipid oxidation, peroxide value, polymers, tocopherols

## Abstract

Conjugated linoleic acid (CLA) is a mixture of positional and geometric isomers of octadecadienoic acid with conjugated double bounds. Positive health properties have been attributed to some isomers, such as anticarcinogenic activity, antiartherosclerotic effects and reduction of body fat gain. Hence, oils rich in CLA such as Tonalin^®^ oil (TO), normally obtained through alkaline isomerization of safflower oil (SO), an oil rich in linoleic acid (LA), are currently used in functional foods. However, special care must be taken to protect them from oxidation to ensure the quality of the supplemented foods. The objective of this work was to evaluate the oxidation and effectiveness of different tocopherol homologues (α-, γ- and δ-), alone or in combination with synergists (ascorbyl palmitate and lecithin), in TO compared to SO at different conditions, ambient temperature (25 °C) and accelerated conditions in Rancimat (100 °C). The oils, the oils devoid of their antioxidants and the latter containing the antioxidants added were assayed. Results showed great differences between SO and TO in terms of formation of hydroperoxides and polymers and also in the effectiveness of tocopherols to delay oxidation. TO showed higher levels of polymerization and, in general, the effectiveness of tocopherol homologues, alone or in combination with synergists, was also lower in the TO.

## 1. Introduction

Oils rich in conjugated linoleic acid (CLA) are nowadays functional ingredients that are added to a variety of foods due to their potentially beneficial health effects. CLA acronym includes a group of positional and geometric isomers of linoleic acid. Numerous animal studies indicate that CLA, specifically the *cis*-9, *trans*-11 isomer, may influence diverse physiological functions and promote health with regard to cancer, atherosclerosis, bone formation, growth modulation and immunity [1]. CLA-rich oils are obtained mainly from alkaline isomerization of linoleic acid-rich oils such as sunflower, soybean and safflower oils [2], since CLA isomers occur naturally only in very minor amounts in food from ruminants, milk and meat. Commercial CLA-rich oils consist almost entirely (*i.e.*, >80%) of the two biologically active CLA isomers (*cis*-9, *trans*-11 and *cis*-10, *trans*-12 linoleic acids) in approximately equal amounts (about 45% each). Although there is no evidence that consumption of such CLA preparations could induce adverse effects in healthy humans [3], safety concerns regarding the use of CLA persist and need further investigation [4].

Despite the growing interest and consumption of CLA-rich oils, scarce information is available on their oxidative stability and effectiveness of antioxidants [5]. However, it is essential to gain insight into the occurrence of oxidised compounds in commercial CLA preparations, since they could lead to adverse physiological effects in cardiovascular and cancerous processes [6], which are precisely the same targets for potential health benefits of CLA.

Studies on CLA oxidation are often contradictory. Oxidation pathways of CLA are still unclear and the mechanisms involved remain controversial [7,8,9,10,11,12,13,14]. Some authors [7,8,11,12,13] have suggested that, in contrast to the case of linoleate, hydroperoxides are only minor products in the oxidation of conjugated linoleate. Therefore, the methods normally used to control lipid oxidation in foods, e.g., peroxide value and thiobarbituric acid reactive substances, may not indicate the real level of oxidation [15,16] and provide misleading results when comparing oxidative stability of CLA and LA. This could be in great part due to formation of polymers from the very beginning of the oxidation process [7,11].

Only three studies have been found in relation to the effect of antioxidants in CLA-rich oils. Effectiveness of tocopherol homologues (α-, β-, γ- and δ-tocopherol) has been studied in CLA at 50 °C through analyses of peroxide value and TBARS, concluding that δ- and γ-tocopherol exhibited the highest activity, whereas α-tocopherol had the lowest protective effect [17]. Likewise, as compared to the synthetic antioxidants TBHQ, BHA and BHT and also to gallic acid, α-tocopherol rendered lower protection in CLA at 45 °C [18]. To our knowledge, the only published work focused on CLA-rich oils tested α-tocopherol in an oil devoid of antioxidants and concluded that it increased its stability to a level similar to that of the LA-rich oil [16]. In this case, results were obtained through determination of the remaining unoxidised substrate and TBARS. The authors found that loss of substrate in CLA-rich oils was significantly greater than that expected from the data obtained for TBARS. This finding could be accounted for by the formation of polymers, as we have already reported in kinetic studies on oxidation of methyl conjugated linoleate [11].

The objective of this work was to evaluate the oxidation and effectiveness of different tocopherol homologues, alone or in combination with synergists (ascorbyl palmitate and lecithin), in a CLA-rich oil, *i.e.*, Tonalin^®^ oil (TO). Different conditions, ambient temperature (25 °C) and accelerated conditions in Rancimat (100 °C), were applied. For comparative purposes, a LA-rich oil, *i.e.*, safflower oil (SO), was also studied. Formation of primary oxidation products, through peroxide value determination, and advanced oxidation products, through quantification of polymers, was monitored in all assays. The intact oils, the oils devoid of their antioxidants and the latter containing α-, γ- or δ-tocopherol added, alone or in combination with both ascorbyl palmitate and lecithin, were studied.

## 2. Experimental Section

### 2.1. Materials and Samples

Tonalin^®^ TG 80 oil (TO) was acquired from Cognis Nutrition & Health (Cincinnati, OH, USA), and refined safflower oil (SO) was purchased from Interfat S.A. (Barcelona, Spain). TO and SO were passed over aluminum oxide (activated at 200 °C for 3 h) column twice in order to remove tocopherols and minor polar compounds as described by Yoshida and coworkers [19]. The tocopherols (α-, γ- and δ-) (purity ≥ 99%), ascorbyl palmitate and lecithin (APL) were purchased from Sigma Chemical Co. (St. Louis, MO, USA). α-, γ- or δ- Tocopherol (500 mg/kg), alone or in combination with ascorbyl palmitate (300 mg/kg) and lecithin (5000 mg/kg), were added to the oils stripped of their naturally occurring antioxidants. Chemicals and reagents used were of analytical grade and obtained from local suppliers.

### 2.2. Oxidation Procedures

Assays under Rancimat conditions: Oil Stability Index (OSI) of TO and SO (starting oils, oils devoid of antioxidants and the latter containing tocopherols added, alone or with APL) was obtained in a Rancimat device (Metrohm, Herisau, Switzerland) at 100 °C with 20 L/h air flow using samples of 2.5 ± 0.1 g [20]. The OSI values thus obtained, as well as the conductivity reading provided by the device, were used in subsequent experiments to carry out appropriate samplings for satisfactory follow-up of oxidation along the test. Special attention was paid to take samples at the end of the induction period (IP), *i.e.*, at times equal to the OSI point. Experiments were done in triplicate. In samples containing antioxidants added, the protection factor was calculated as the ratio between the OSI values in the presence and absence of antioxidants.

Assays at ambient temperature: Five-gram samples of TO and SO (starting oils and oils devoid of antioxidants) were placed in 1 L-glass beakers. The beakers were placed at a temperature-controlled chamber and stored at 25 ± 3 °C in the dark. Aliquots (100 mg) were withdrawn for analyses along the oxidation experiment. Experiments were done in triplicate.

### 2.3. Analytical Methods

#### 2.3.1. Determination of Tocopherols

Tocopherols were determined by normal-phase HPLC with fluorescence detection according to IUPAC Standard Method 2.411 [21].

#### 2.3.2. Quantitation of Triacylglycerol Dimers and Higher Oligomers by High-Performance Size-Exclusion Chromatography (HPSEC)

Aliquots of 50 mg oil were dissolved in 1 mL tetrahydrofuran for direct analysis by HPSEC. A chromatograph equipped with a Rheodyne 7725i injector (Hamilton, NV, USA) with a 10-μL sample loop, a Knauer 120 HPLC pump (Knauer, Berlin, Germany) and a Merck L-7490 refractive index detector (Merck, Darmstadt, Germany) was used. The separation was performed on two 100 and 500 Å Ultrastyragel columns (25 cm × 0.77 cm ID, Hewlett-Packard, Avondale, PA, USA) packed with porous, highly cross linked styrene-divinylbenzene copolymers (particle size: 5 μm) (Hewlett-Packard, Avondale, PA, USA) connected in series, with tetrahydrofuran (1 mL/min) as the mobile phase according to IUPAC Standard Method 2.508 [21]. The groups of compounds quantified were dimers and higher oligomers [22]. The sum of dimers and higher oligomers will be referred to as polymers.

#### 2.3.3. Determination of the Peroxide Value (PV)

Peroxide value was determined by the iodometric assay following IUPAC standard method 2.501 [21].

#### 2.3.4. Analysis of Fatty Acid Composition

Fatty acid composition was determined by GC-FID analysis. The oils were converted into fatty acid methyl esters (FAME) using 2 M KOH in methanol (IUPAC, 1992). FAME were analysed on an HP-6890 chromatograph (Hewlett Packard, Avondale, PA, USA) equipped with a split/splitless injector and a FID detector. Fatty acids were separated using an HP Innowax capillary column (30 m × 0.25 mm ID × 0.25 μm film thickness, Hewlett Packard, Avondale, PA, USA). The column was held at 180 °C for 2 min after injection, temperature-programmed at 3 °C/min to 230 °C and held there for 20 min. A split ratio of 1:40 was applied and hydrogen was used as carrier gas (1 mL/min). The injector temperature was set at 250 °C and detector temperature was set at 270 °C.

### 2.4. Statistical Analysis

Data of fatty acid composition, tocopherols, peroxide value, polymers and OSI in the starting oils, as well as OSI in the oils devoid of their antioxidants and with tocopherols and synergists added, were obtained by using three determinations. The oxidation experiments were carried out in triplicate and results for polymers and peroxide value have been expressed as mean values. Comparisons between means were made by applying one-way ANOVA using SPSS Statistics version 17.0 (SPSS Inc., Dublin, Ireland). Differences between means were determined using post-hoc Tukey’s test. Significant differences were established at *p* < 0.05.

## 3. Results and Discussion

Table 1 shows fatty acid and tocopherol compositions and oxidative parameters of SO and TO. The content of linoleic acid (C18:2 9c, 12c) in SO was similar to that of total CLA (C18:2 9c, 11t and C18:2 10t, 12c) in TO. Even though TO contained approximately twice as much of total tocopherols as SO, the two oils presented similar OSI values, 5.8 and 5.7 h for SO and TO, respectively. The PV and polymers were low in both oils and typical for fresh refined oils.

**Table 1:** Composition and oxidative parameters of Safflower and Tonalin^®^ oils.

Parameter	Safflower Oil	Tonalin Oil
Fatty acid composition (%)		
16:0	7.2 ± 0.2	2.4 ± 0.1
18:0	2.6 ± 0.1	2.6 ± 0.1
18:1	13.7 ± 0.5	14.2 ± 0.6
18:2 *9c*, *12c*	74.7 ± 0.7	0.5 ± 0.1
18:2 *9c*, *11t* (CLA)		38.2 ± 0.7
18:2 *10t*, *12c* (CLA)		38.6 ± 0.7
Others	1.8 ± 0.1	3.5 ± 0.2
Tocopherols (mg/kg)		
α	266 ± 13	28 ± 2
γ		324 ± 17
δ		215 ± 13
Peroxide value (meq O_2_/kg)	2.9 ± 0.2	2.5 ± 0.3
Oil Stability Index (h)	5.8 ± 0.4	5.7 ± 0.3
Polymers (%)	1.0 ± 0.3	1.1 ± 0.4

Data are expressed as Means ± Standard Deviations (*n* = 3). SO: safflower oil. TO: Tonalin^®^ oil; *c*: *cis*; *t*: *trans*; CLA: conjugated linoleic acid.

**Figure 1:**
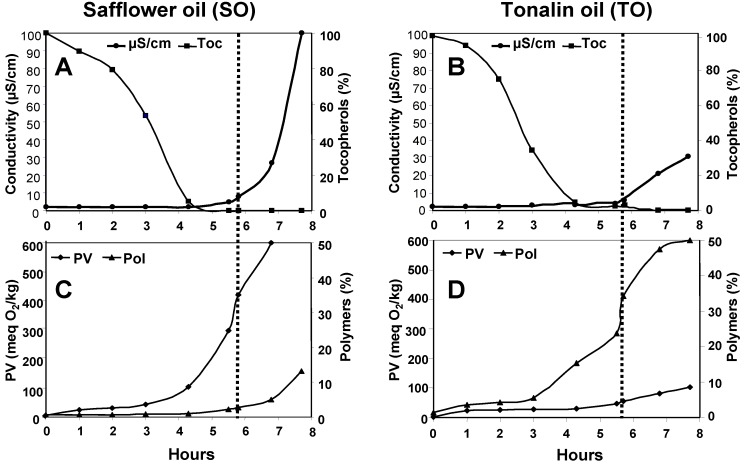
Oxidation of Safflower (**A**,**C**) and Tonalin (**B**,**D**) oils at 100 °C in Rancimat. Data are expressed as Means (*n* = 3 experiments). Coefficients of variation were equal to or lower than 8% for peroxide values and tocopherols, and 5% for polymers. PV: peroxide value, POL: polymers, Toc: tocopherols.

Figure 1 shows the progress of different parameters during oxidation in Rancimat at 100 °C. In both oils, the end of the induction period or OSI value (dotted lines) was marked by the exhaustion of tocopherols (Figure 1A,B). At that point, the accelerated oxidation stage starts and a rapid increase in conductivity is normally obtained [23]. However, TO showed a slower increase in conductivity after the end of the induction period, thus indicating slower formation of volatile compounds detected by conductivity changes.

The most relevant differences between the oils were observed in the progress of PV and polymers. As expected, SO showed a progressive increase in the PV from the onset of the assay, reaching a value of 421 meq O_2_/kg at the end of the induction period. Until then, the polymer content had not practically increased (2.1%), but exhaustion of tocopherols triggered polymerization beyond that point. Quite in contrast, TO showed the opposite oxidative behaviour, *i.e.*, a marked increase in polymers during the induction period, reaching values as high as 32.9% at the end and comparatively low PV levels (56 meq O_2_/kg). The results obtained at 100 °C indicate that polymerization is favoured in TO at early stages of oxidation and tocopherols were not capable of preventing it.

**Figure 2:**
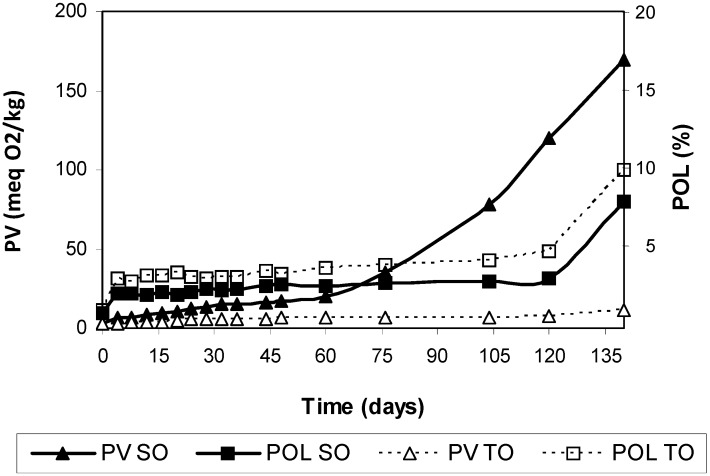
Peroxide values and polymer concentrations during storage of safflower oil (SO) and Tonalin oil (TO) at 25°C in the dark. Data are expressed as Means (*n* = 3 experiments). Coefficients of variation were equal to or lower than 8% for peroxide values and 5% for polymers. PV: peroxide value, POL: polymers.

With the aim of comparing SO and TO under realistic storage conditions, assays were carried out at 25 °C in the dark. Table 2 and Table 3, and Figure 2 include the results obtained.

**Table 2:** Oxidation of safflower oil at 25 °C.

Days	Dimers (%)	Oligomers (%)	Total Polymers (%)	Peroxide Value (meq O_2_/kg)	Tocopherols (mg/kg)
0	1.0	0.0	1.0	2.9	266
4	2.0	0.2	2.2	6.9	192
8	1.9	0.2	2.2	6.3	179
12	1.9	0.2	2.1	8.4	174
16	2.1	0.2	2.3	9.9	150
20	1.9	0.2	2.1	10.0	139
24	2.0	0.3	2.3	12.8	139
28	2.1	0.4	2.5	13.3	118
32	2.1	0.3	2.4	15.0	100
36	2.1	0.3	2.4	15.7	80
44	2.3	0.4	2.7	16.2	42
48	2.3	0.4	2.8	17.6	40
60	2.3	0.4	2.7	20.3	33
76	2.4	0.4	2.8	35.3	29
104	2.5	0.4	2.9	77.9	18
120	2.7	0.5	3.2	120.0	11
140	5.9	2.1	8.0	170.0	0

Data are expressed as Means (*n* = 3 experiments). Coefficients of variation were equal to or lower than 8% for peroxide values and tocopherols, and 5% for dimers, oligomers and total polymers.

**Table 3:** Oxidation of Tonalin oil at 25 °C.

Days	Dimers (%)	Oligomers (%)	Total Polymers (%)	Peroxide Value (meq O_2_/kg)	Tocopherols (mg/kg)
0	0.0	1.1	1.1	2.5	568
4	0.2	2.9	3.2	2.9	532
8	0.2	2.7	2.9	3.3	525
12	0.2	3.1	3.4	4.0	480
16	0.2	3.1	3.4	4.0	445
20	0.3	3.3	3.6	4.9	446
24	0.2	3.0	3.3	5.3	446
28	0.2	2.9	3.1	5.7	442
32	0.2	3.1	3.3	5.6	429
36	0.2	3.0	3.2	5.9	377
44	0.2	3.4	3.7	5.8	358
48	0.2	3.3	3.4	6.3	357
60	0.2	3.6	3.8	6.5	322
76	0.2	3.8	4.0	6.9	275
104	0.2	4.1	4.3	7.1	180
120	1.6	3.3	4.9	7.8	107
140	1.8	7.2	10.0	11.0	0

Data are expressed as Means (*n* = 3 experiments). Coefficients of variation were equal to or lower than 8% for peroxide values and tocopherols, and 5% for dimers, oligomers and total polymers.

The end of the induction period in SO was around 120 days, as defined by the start of significant polymerization, PV above 100 meq O_2_/kg and only negligible amounts of tocopherols remaining. This oxidation pattern has been repeatedly observed in our previous studies on oils and model triacylglycerols [24,25,26]. However, the induction period was far from clear in the case of TO. Following also a total depletion of tocopherols after 120 days of storage, polymers increased more rapidly although their formation had started from the beginning of the assay, while PV changes were small and a level as low as 11 meq O_2_/kg was reached at the end of the experiment.

The pool of polymers in TO comprised trimers and larger oligomers only (Table 3). In fact, the increase in polymers in TO throughout oxidation was essentially attributable to the increase of large oligomers. We already found a large degree of polymerization in a previous work comparing oxidation kinetics of conjugated methyl linoleate and non-conjugated methyl linoleate under mild oxidation conditions [11]. Similarly to the results obtained here in CLA-rich oils, conjugated methyl linoleate showed a very different oxidation pattern from that of non-conjugated methyl linoleate. Firstly, formation of typical primary oxidation products, *i.e.*, hydroperoxides, seemed to be negligible, secondly, dimer formation was rare and, thirdly, the starting point of oxidation was characterized by the appearance of polymers of unusually high molecular weight or size. These results indicate that formation of very complex molecules is favored even under the mild oxidation conditions used, *i.e.*, 25 °C in the dark.

**Figure 3:**
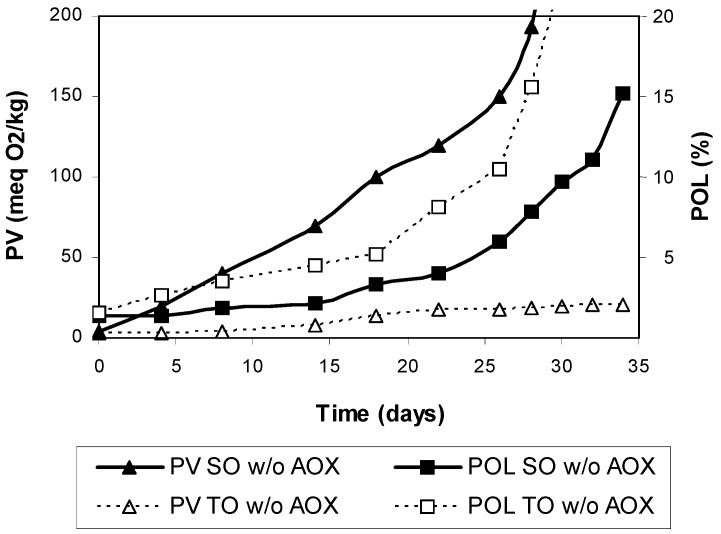
Peroxide values and polymer concentrations during storage of antioxidant-stripped safflower oil (SO w/o AOX) and Tonalin oil (TO w/o AOX) at 25 °C in the dark. Data are expressed as Means (*n* = 3 experiments). Coefficients of variation were equal to or lower than 8% for peroxide values and 5% for polymers. PV: peroxide value, POL: polymers.

For comparative purposes, the antioxidant-stripped oils were also tested at 25 °C. Clearly, polymer formation was greater in TO than in SO along oxidation and marked differences were observed in the PV (Figure 3). For example, the PV was over 200 meq O_2_/kg in SO at 30 days of storage, while it was only 19 meq O_2_/kg in TO.

It is well known that the mechanism of autoxidation of methylene-interrupted fatty acid double bonds involves a catalytic process which proceeds via a free radical mechanism [27]. The initiation step consists of alkyl radical formation by the abstraction of a hydrogen radical from the carbon adjacent to the double bond. In the propagation step, oxygen is added to form alkylperoxyl radicals and the oxygen consumed is primarily converted to hydroperoxides. However, the low occurrence of hydroperoxides and high polymerization previously observed in CLA model compounds [11] and in CLA-rich oils in the present work support that different mechanisms could be involved. Yurawecz and coworkers [8,9,10,11,12] suggested that the free radical mechanism is not likely to occur in conjugated fatty acids because high energy is previously required to separate double bonds from conjugation. Therefore, formation of oxidation compounds other than those expected from the free radical mechanism may be favoured. The authors reported that CLA underwent 1,2- and 1,4-cycloadditions with oxygen, which gave rise to dioxetane structures and endoperoxides leading to furan fatty acids, respectively. Other reactions such as dimerization and polymerization, although not evaluated, were not ruled out by the authors. In this regard, pioneering studies on conjugated polyunsaturated fatty acids, specifically β-eleostearic acid, already established that oxygen-containing polymers were main oxidation products [28]. Later, comparative oxidation studies on 9,12- and 10,12-methyl linoleate suggested that carbon-to-oxygen polymerization occurred rather than carbon-to-carbon polymerization [29] and that the peroxide decomposition is not a major factor in the mechanism of oxidation of conjugated substances [30]. Recently, based upon bond dissociation energy values, Oyman and coworkers [31] have suggested that the preferred reaction pathway for a free radical in tung oil, a drying oil containing substantial contents of the conjugated triene fatty acid α-eleostearic acid, is the addition to a conjugated double bond rather than abstracting a monoallylic hydrogen like in the propagation step of the oxidation of non-conjugated unsaturated fatty acids. By comparison with linseed oil, a drying oil containing substantial contents of the non-conjugated triene fatty acid α-linolenic acid, these authors observed lower absorption of oxygen and, in agreement with the results obtained for SO and TO in the present study, much lower PV in tung oil.

**Figure 4:**
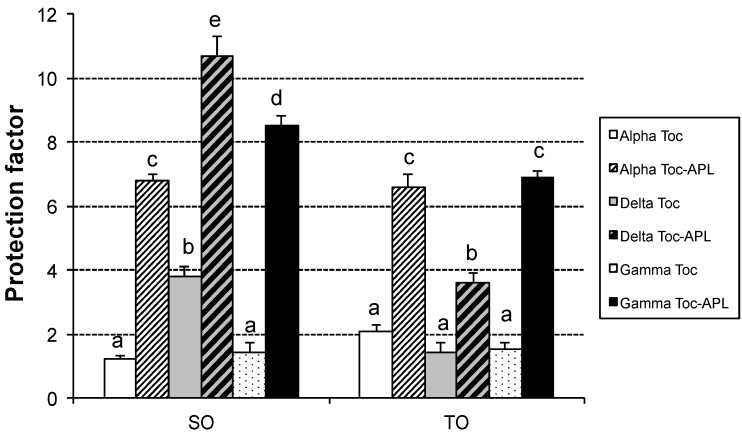
Protection factors (ratio between the oil stability index in the presence and absence of antioxidants) provided by tocopherol homologues alone and in combination with APL in safflower oil (SO) and Tonalin oil (TO) stripped of their naturally occurring antioxidants in Rancimat at 100 °C. Data are expressed as Means ± Standard Deviations (*n* = 3). In each oil, different letters mean significant differences (*p* < 0.05). Toc: Tocopherol; APL: Ascorbyl Palmitate and Lecithin.

In order to examine the effectiveness of tocopherol homologues, alone and in combination with APL, the SO and TO stripped of their naturally occurring antioxidants were used and assays were carried out in Rancimat at 100 °C. OSI values for the antioxidant-stripped SO and TO were rather low, 2.2 and 2.8 h, respectively. Figure 4 represents the protection factors provided by the antioxidants tested.

In general, TO seemed less protected than SO by the antioxidants used, especially the samples containing tocopherol along with APL added. In the case of SO, α-tocopherol and α-tocopherol with APL were the least protected samples as compared with their counterparts, while δ-tocopherol and δ-tocopherol with APL were the most effective antioxidants. For the most stable samples, *i.e.*, those containing tocopherol along with APL, the order of antioxidant effectiveness was δ- > γ- > α-. These results are in agreement with other studies that show an order of antioxidant effectiveness in the opposite direction to the hydrogen-donating power, *i.e.*, δ- > γ- > α-, in fats and oils under different experimental conditions [32,33,34,35]. For the same substrate, concentration and oxidation conditions, the relative activities of the tocopherols are not only dependent on their chemical reactivity toward peroxyl and other free radicals, but also on many other possible side reactions that are likely to affect α-tocopherol to a greater extent [36]. As to the synergistic combination used, it has also been reported that the antioxidant effect of tocopherols greatly improves by addition of ascorbyl palmitate in polyunsaturated oils [37,38], being able to regenerate tocopherol.

As to the TO samples, any of the individual tocopherols studied provided a poor protection, which was similar for the three antioxidants. Unlike the SO, results for the combination of tocopherols with APL showed the lowest protection factor for δ-tocopherol and the highest for α- and γ-tocopherol, being similar for both.

The effectiveness of tocopherol homologues, α-, β-, γ- or δ-tocopherol, on CLA has recently been studied at 50 °C by Ko and coworkers [17]. The results showed that δ- and γ-tocopherol exhibited the highest activity, whereas α-tocopherol had the lowest protective effect. Nevertheless, the results were obtained through analyses of PV and TBARS only. A misleading picture of the development of oxidation of CLA could have been obtained, given that hydroperoxides (PV) or thio-barbituric acid reactive substances, mainly malondialddehyde, are not the major oxidation compounds formed in CLA oxidation. To our knowledge, similar studies on CLA-rich oils have not yet been published.

## 4. Conclusions

The results obtained in this study reflect that different oxidation pathways could be involved in LA-rich and CLA-rich oils, the latter showing an oxidation pattern in which polymers are the main group of compounds formed. It appears that polymer quantitation can be an appropriate measurement to follow-up oxidation of CLA-rich oils *vs.* peroxide value, which is at present the only determination included in the quality specifications in relation to oil oxidation level. Also, the effectiveness of tocopherols combined with APL in TO, usually found in CLA-supplemented foods, has been found to be much lower than expected, thus demanding further research on antioxidant mode of action and efficiency in CLA-rich oils.
